# Comparing efficacy and safety of stair step protocols for clomiphene citrate and letrozole in ovulation induction for women with polycystic ovary syndrome (PCOS): a randomized controlled clinical trial

**DOI:** 10.25122/jml-2023-0069

**Published:** 2023-05

**Authors:** Saba Al-Thuwaynee, Asmaa Abdul Jaleel Swadi

**Affiliations:** 1. Department of Obstetrics and Gynecology, College of Medicine, University of Al-Qadisiyah, Al Diwaniyah, Iraq; 2. Department of Pharmacology, College of Medicine, University of Al-Qadisiyah, Al Diwaniyah, Iraq

**Keywords:** efficacy, safety, clomiphene citrate, letrozole, polycystic ovary syndrome

## Abstract

Polycystic ovary syndrome (PCOS) is characterized by menstrual irregularities, high androgen levels, and ovarian cysts. Clomiphene citrate (Clomid) and letrozole have both been investigated as ovulation induction therapies for PCOS. This interventional study aimed to compare the efficacy and safety of a stairstep practice of letrozole versus clomiphene citrate in women with PCOS. A total of 100 women diagnosed with PCOS and infertility participated in the study, which took place from March 2021 to July 2022 at the Maternity and Children Teaching Hospital in Adiwaniyah Province, Iraq. Participants were randomly assigned to one of two groups (each with 50 women): the first group received clomiphene citrate in a stair step pattern (single dose of 50 mg, 100 mg, and 150 mg) for five days, for a maximum of three cycles; the second group received letrozole in a stair step pattern (single dose of 2.5, 5, and 7.5 mg) for five days, for a maximum of three cycles. Follicle size was monitored using ultrasound to achieve a follicle size >18 mm. The ovulation rate was higher in the letrozole group (86.0%) compared to the clomiphene citrate group (72.0%), although the difference was not statistically significant (p=0.086). The pregnancy rate was slightly higher in the letrozole group (22.0% *vs* 18.0%), but also not statistically significant (p=0.617). However, the mean time from menstruation to ovulation was significantly shorter in the letrozole group (17.20±1.32 days) compared to the clomiphene citrate group (24.08 ± 1.56 days, p<0.001). There were no significant differences in common side effects between the two groups. Overall, letrozole was found to be as safe as clomiphene citrate but demonstrated a shorter time to ovulation. Further studies with larger sample sizes are necessary to validate these findings and determine their clinical implications.

## INTRODUCTION

Polycystic ovary syndrome (PCOS) is a complex clinical condition characterized by menstrual irregularities, high levels of androgen, and cystic changes in the ovaries [1,2]. The manifestations of PCOS can vary, with some cases primarily driven by hyperandrogenemia, leading to a biochemical dominance, while others are primarily characterized by polycystic ovaries, resulting in a morphological dominance [3]. The disease affects approximately 7% of women of reproductive age [4], imposing a substantial economic burden of approximately $4 billion per year in the United States alone [5]. It is the most common endocrine disorder affecting women between the ages of 18 to 45 years [6].

PCOS is frequently diagnosed in women presenting with infertility, excessive hair growth, amenorrhea, acne, and obesity [7]. Moreover, women with PCOS have higher rates of type 2 diabetes mellitus, abnormal lipid metabolism, cardiovascular disease, and endometrial malignancy compared to women without the condition [8,9]. The etiology of PCOS is complex and is believed to involve the interaction between genetic predisposition and environmental factors [10-12]. While a positive family history suggests genetic predisposition, the exact mode of inheritance remains unclear, despite some claims of a near autosomal dominant mode [12]. Environmental factors such as poor dietary habits and lack of exercise predispose to obesity, which has been linked to the disease by several authors, in addition to infections and toxins. Weight loss and exercise have been shown to reverse some of the biochemical and reproductive drawbacks of the disease [3].

One of the primary pharmacological agents utilized to induce ovulation in the treatment of PCOS, is clomiphene citrate (Clomid) [13]. The agent can be used repeatedly in successive cycles, with a maximum of 6 cycles, aiming to achieve a successful pregnancy. In cases where pregnancy is not achieved, alternative medical approaches are considered. It has been shown that clomiphene citrate can result in pregnancy in approximately 30% of cases. However, it is worth mentioning that around 20% of these pregnancies may end in spontaneous abortions [3]. The main side effects of this agent are ovarian hyperstimulation syndrome (OHSS), enlargement of ovaries, hot flashes, gastrointestinal upset and multiple pregnancies [14].

Letrozole, primarily known for its use in certain breast cancer cases, has also been explored as an effective ovulation inducer [15]. The standard approach to letrozole for ovulation stimulation is similar to the strategy employed with clomiphene citrate [16]. The treatment protocol typically begins with the lowest dose of letrozole administered for 5 days, starting on cycle day 3–5 following either a spontaneous menstrual cycle or a progesterone-induced withdrawal bleed. In cases when ovulation is not detected, confirmed through mid-luteal progesterone levels or ultrasonography, the patient receives progesterone to stimulate withdrawal bleeding, mimicking a normal menstruation. The dosage is then incrementally increased in a systematic manner with each subsequent cycle until the maximum dose of letrozole is reached.

The “stair step” protocol eliminates the use of progestin to induce a withdrawal bleed between successive treatment cycles. By eliminating the progestin withdrawal step, the time to ovulation is reduced, allowing for a more rapid administration of the ovulation-inducing medication. Stair-step procedures using clomiphene citrate (CC) have been extensively studied for ovulation stimulation [17,18]. The first description of stair-step procedure using letrozole in PCOS was made by Thomas *et al*. [19] in 2019. This study aimed to evaluate the efficacy and safety of the stair-step procedure using letrozole compared to that of clomid in women diagnosed with polycystic ovary syndrome.

## MATERIAL AND METHODS

### Study design and location

**Figure 1.**
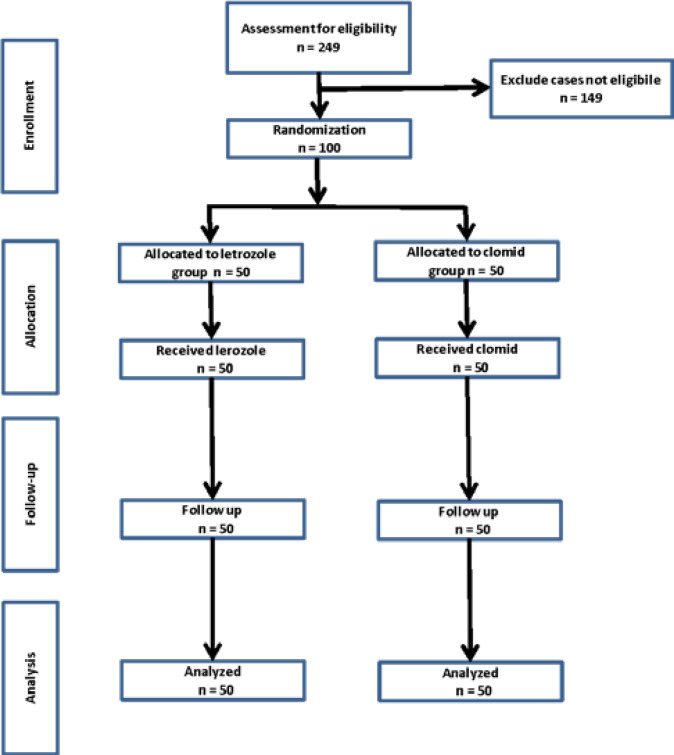
Flowchart showing study design based on CONSORT guidelines

The study was designed as an interventional trial, and patients were randomly allocated using a simple randomization technique. The intervention followed a parallel assignment model, and the study was conducted in an open-label manner with no masking. The primary objective of the study was to evaluate the efficacy of the treatment. The research took place at the Maternity and Children Teaching Hospital in Adiwaniyah Province, Iraq. The study procedures adhered to the guidelines outlined in the CONSORT statement, as depicted in Figure 1.

### Enrollment

The study enrolled 100 women diagnosed with polycystic ovarian syndrome (PCOS) based on the 2003 Rotterdam criteria. Inclusion criteria required the presence of oligo/anovulation and either medical or biochemical manifestations of hyperandrogenism, polycystic ovaries, or oligoovulation/anovulation, along with infertility (defined as the inability to conceive after one year of unprotected intercourse), while excluding other causes of androgen excess, infertility, and the use of medications for ovulation induction other than letrozole or CC during the specified period.

### Allocation, treatment protocol and follow-up

Women were randomly allocated into two groups, with each group consisting of 50 participants. The first group underwent the stair-step pattern of ovulation induction using clomid at a dose of 50 mg (single daily dose) from day two of the cycle until day 6 (spontaneous or induced menses). The same stair-step pattern was followed in the second group, but they received letrozole at a dose of 2.5 mg (once daily) from day 2 of the cycle for 5 days. Ultrasound examination was conducted 6 to 7 days after the last dose of the drug and if follicle size was ≥ 18 mm, 10,000 IU hCG was administered for ovulation induction.

In cases where no follicle reaction was observed (all follicles <10 mm) or the single follicle size was <18 mm, the daily drug dose of either clomid or letrozole was immediately doubled without withdrawal bleeding and given for another 5 days. Subsequent ultrasound examinations were conducted 7 days after the last dose. If the desired follicle size of >18 mm was not achieved, the process was repeated by tripling the initial dose of clomid or letrozole, with maximum doses of 150 mg and 7.5 mg, respectively, and hCG was administered accordingly.

Ovulation was considered successful when confirmed by a positive ovulation test kit or documented ultrasound evidence of a follicle of at least 18 mm size that was likely to ovulate. The treatment protocol was repeated for three cycles on the same menstrual cycle, depending on the follicle size. The study started in March 2021 and ended in July 2022. Data on age, body mass index (BMI), parity, thyroid stimulating hormone level, fasting blood sugar and prolactin level were collected. Women were followed up to assess overall ovulation rate, pregnancy rate and mean time from menstruation to ovulation. Safety was assessed based on the frequency of nausea, vomiting, headache, dizziness and blurred vision.

### Body mass index calculation

BMI was calculated by dividing the weight in kilograms by the square of height in meters.

### Blood chemistry

Fasting blood samples were collected for glucose, prolactin, and thyroid-stimulating hormone (TSH) measurements after a 10-12 hour fasting period. The chemical analysis was carried out using the Beckman Coulter autoanalyzer AU2700.

### Statistical analysis

Data obtained from the study were entered into an SPSS spreadsheet (version 16) for statistical description and analysis. Categorical variables were presented as percentages and numbers, while quantitative data were expressed as range, standard deviation, mean, median and inter-quartile range. For comparisons of proportions, either the Yates correction, Chi-square test, or Fisher's exact test was employed based on the statistical assumptions that justified each analysis. The level of significance was set at p≤0.05.

## RESULTS

Table 1 presents the general characteristics of the PCOS patients enrolled in the study. The mean age of all enrolled women was 30.09±3.35 years, with an age range of 24 to 37 years. There was no significant difference in the mean age between the clomid group and the letrozole group (p=0.458). The mean body mass index (BMI) of all enrolled women was 28.95 ±3.26 kg/m2, with a range of 21 to 35 kg/m2. There was no significant difference in the mean BMI between the two treatment groups (p=0.134). The study included patients with a parity range of 0 to 3, and the median parity was 1 (interquartile range of 2) across all enrolled participants. The comparison between the clomid group and the letrozole group revealed no statistically significant difference in parity (p=0.208).

**Table 1.** Demographic characteristics of participants

Characteristic	Total n = 100	Clomid group n = 50	Letrozole group n = 50	P
**Age (years)**				
Mean ±SD	30.09±3.35	29.84±2.97	30.34±3.70	0.458 I NS
Range	24 -37	25 -35	24 -37	
**BMI (kg/m^2^)**				
Mean ±SD	28.95±3.26	28.46±3.28	29.44±3.21	0.134 I NS
Range	21 -35	21 -35	25 -35	
**Parity**				
Median (IQR)	1 (2)	1 (2)	1 (2)	0.208 M NS
Range	0 -3	0 -3	0 -2	

Table 2 presents the comparison of fasting blood sugar and hormonal levels between the clomid group and the letrozole group. The mean fasting blood sugar level among all participants was 87.43±8.36 mg/dl. In the clomid group, the mean fasting blood sugar level was 90.28±6.29 mg/dl, while in the letrozole group, it was 84.58±9.22 mg/dl. The ranges for fasting blood sugar were 71 to 100 mg/dl, 80 to 100 mg/dl, and 71 to 100 mg/dl, respectively. There was a significant difference in fasting blood sugar levels between the two groups (p<0.001).

**Table 2.** Fasting blood sugar and hormonal levels in the Clomid and Letrozole groups

Characteristic	Total n = 100	Clomid group n = 50	Letrozole group n = 50	P
**FBS (mg/dl)**				
Mean ±SD	87.43±8.36	90.28±6.29	84.58±9.22	< 0.001 I ***
Range	71 -100	80 -100	71 -100	
**TSH (mIU/L)**				
Mean ±SD	2.39±1.71	2.44±1.67	2.35±1.77	0.799 I NS
Range	0 -5	0 -5	0 -5	
**Prolactin (ng/ml)**				
Mean ±SD	14.40±7.07	14.92±6.81	13.88±7.35	0.465 I NS
Range	2 -25	2 -25	2 -25	

The mean TSH of all enrolled women was 2.39±1.71 mIU/L, with no significant differences between the two groups (p=0.799). The mean serum prolactin level was 14.40±7.07 ng/ml in all enrolled women, with no significant differences between the clomid group and the letrozole group (p=0.465).

Table 3 presents the evaluation of response to treatment between the clomid group and the letrozole group. The ovulation rate was higher in the letrozole group compared to the clomid group, (86.0% vs. 72.0 %), although the difference was not statistically significant (p=0.086). Similarly, the pregnancy rate was slightly higher in the letrozole group compared to the clomid group (22.0% vs. 18.0%), but the difference was not significant (p=0.617). The mean time from menstruation to ovulation among all participants was 20.64±3.75 day and it was significant shorter in the letrozole group compared to the clomid group (17.20±1.32 days vs. 24.08±1.56 days, p<0.001).

**Table 3.** Response to treatment in the two groups

Characteristic	Total n = 100	Clomid group n = 50	Letrozole group n = 50	P
**Overall ovulation rate**				
Negative, n (%	21 (21.0 %)	14 (28.0 %)	7 (14.0 %)	0.086 C NS
Positive, n (%)	79 (79.0 %)	36 (72.0 %)	43 (86.0 %)	
**Pregnancy rate**				
Negative, n (%)	80 (80.0 %)	41 (82.0 %)	39 (78.0 %)	0.617 C NS
Positive, n (%)	20 (20.0 %)	9 (18.0 %)	11 (22.0 %)	
**Mean time of ovulation day**				
Mean ±SD	20.64±3.75	24.08±1.56	17.20±1.32	< 0.001 I ***
Range	15 -27	22 -27	15 -20	

Table 4 provides a detailed comparison of the side effects reported by participants in the clomid group and the letrozole group during the course of treatment. The rates of side effects among all enrolled PCOS women were as follows: nausea and vomiting (5.0%), headache (5.0%), dizziness (5.0%), and blurred vision (1.0%). Importantly, there was no significant difference in the rates of these side effects between the clomid group and the letrozole group (p>0.05). These findings suggest that the incidence of these side effects, represented by similar percentages in both treatment groups was comparable.

**Table 4.** Treatment side effects in the two groups

Characteristic	Total n = 100	Clomid group n = 50	Letrozole group n = 50	P
**Nausea and vomiting**				
Negative, n (%)	95 (95.0 %)	47 (94.0 %)	48 (96.0 %)	1.000 Y NS
Positive, n (%)	5 (5.0 %)	3 (6.0 %)	2 (4.0 %)	
**Headache**				
Negative, n (%)	95 (95.0 %)	48 (96.0 %)	47 (94.0 %)	1.000 Y NS
Positive, n (%)	5 (5.0 %)	2 (4.0 %)	3 (6.0 %)	
**Dizziness**				
Negative, n (%)	95 (95.0 %)	47 (94.0 %)	48 (96.0 %)	1.000 Y NS
Positive, n (%)	5 (5.0 %)	3 (6.0 %)	2 (4.0 %)	
**Blurred vision**				
Negative, n (%)	99 (99.0 %)	49 (98.0 %)	50 (100.0 %)	1.000 F NS
Positive, n (%)	1 (1.0 %)	1 (2.0 %)	0 (0.0 %)	

**n:** number of cases; **Y:** Yates correction test; **F:** Fischer exact; **NS:** not significant

## DISCUSSION

The use of letrozole, an aromatase inhibitor, for inducing ovulation in women with anovulatory infertility has been well established [20,21]. Letrozole has shown efficacy in cases where clomiphene treatment was ineffective, with ovulation rates reported as high as 62% and conception rates of 14.7% [22, 23]. By suppressing the aromatase enzyme, letrozole inhibits estrogen production, leading to a significant reduction in estrogen levels, reportedly as high as 97% to 99% [24,25]. Additionally, no adverse fetal outcomes have been documented [23]. However, more evidence is needed to conclusively establish the efficacy of letrozole in treating infertility [26]. In the context of treating infertility in women with PCOS, letrozole plays a crucial role. However, its efficacy remains variable, particularly when compared to clomiphene [26]. When compared to clomiphene, several earlier studies found letrozole to be less effective [27, 28]. Letrozole, on the other hand, has been shown to dramatically increase the living birth and pregnancy proportions in PCOS patients in various systematic reviews and meta-analyses [29,30].

Our study findings align with the results of the Thomas *et al*. [19], who compared ovulation induction using the stair-step pattern of letrozole and clomid in reproductive-age women with PCOS. While no significant difference in ovulation rates was observed, the letrozole group showed shorter ovulation time compared to the clomid group, while the pregnancy rates were comparable between the two groups.

In another study by Sakar *et al*. [31], a retrospective comparison was made between clomid-resistant patients treated with the clomid stair-step protocol and a group of patients who were identified as clomid-resistant and subsequently treated with the letrozole stair-step protocol. The results showed significantly higher rates of ovulation, shorter mean time from menstruation to ovulation and greater pregnancy level in the letrozole group compared to the clomid group. While we agree with Sakar *et al*. regarding the timing of ovulation, we disagree with their findings on ovulation and pregnancy rates. A study by Guang *et al*. [26], found no significant differences in adverse events between the letrozole and clomiphene groups. Furthermore, there were no notable differences in the primary endpoints of live birth, birth weight, and infant gender, as well as secondary endpoints such as pregnancy rates and pregnancy loss. This suggests that letrozole and clomiphene are equally effective and safe for treating PCOS-related infertility in women.

A meta-analysis conducted by Tsiami *et al*. [32] compared letrozole and clomid for their ability to induce ovulation in infertile women with PCOS, using scheduled sexual activity or intrauterine fertilization. The analysis showed a significantly higher likelihood of ovulation in letrozole cycles compared to clomiphene cycles. The meta-analysis included 26 randomized controlled trials with 4,168 patients and 8,310 cycles of ovulation stimulation, with studies published between 2006 and 2019.

We acknowledge that the current study had a limitation related to the enrollment of participants from multiple centers across the country. Due to communication challenges and limited availability of facilities, it was difficult to include a diverse range of participants from different locations.

## CONCLUSION

Based on our observations, along with the findings reported by previous authors, it can be concluded that letrozole is a safe and comparable alternative to clomid for inducing ovulation in women with anovulatory infertility. Both medications demonstrate similar safety profiles, but letrozole offers the advantage of a shorter time from menstruation to ovulation.
